# Moral dilemmas in females: children are more utilitarian than adults

**DOI:** 10.3389/fpsyg.2015.01345

**Published:** 2015-09-08

**Authors:** Monica Bucciarelli

**Affiliations:** Dipartimento di Psicologia, Centro di Scienza Cognitiva, Università di TorinoTorino, Italy

**Keywords:** moral dilemmas, utilitarianism, intuitions, deliberative reasoning, mental models

## Abstract

Influential theories on moral judgments propose that they rely either on emotions or on innate moral principles. In contrast, the mental model theory postulates that moral judgments rely on reasoning, either intuition or deliberation. The theory allows for the possibility that intuitions lead to utilitarian judgments. This paper reports two experiments involving fifth-grade children, adolescents, and adults; the results revealed that children reason intuitively to resolve moral dilemmas in which action and inaction lead to different outcomes. In particular, the results showed female children to be more utilitarian than female adults in resolving classical moral dilemmas: they preferred an action that achieved a good outcome for a greater number of people. Within the mental model theory's framework there is no reason to expect that females and males differ in their ability to reason, but at the moment the results for females cannot be generalized to males who were not properly represented in the adults groups of the two experiments. The result revealing that (female) children are more utilitarian than (female) adults, which is hard to explain via many current theories, was predicted by the mental model theory.

## Introduction

Moral judgments pervade our daily life; we often engage in considerations on whether an action is morally permissible or impermissible. The assumption underlying my investigation is that moral judgments of this sort are grounded on reasoning. This idea was first introduced in psychology by Piaget (1965/1932) and Kohlberg (1984), who tackled the question of which processes are involved in moral judgment from a developmental perspective. They reported on many dialogs conducted with children illustrating how they reason about what is right and what is wrong. Many of the most recent theories on moral judgments are, instead, concerned with adults and deny a role for reasoning. The socio-intuitionist theory argues in favor of emotions (Haidt, 2001, 2007) and the moral grammar theories argue in favor of innate moral principles (Mikhail, 2000, 2011; Hauser, 2006a). However, some theories on moral judgments underscore the importance of deliberative thought in the process of moral evaluation (see Royzman et al., 2009; Paxton et al., 2012; Pennycook et al., 2014). In particular, the theory of mental models—henceforth, the “model” theory—postulates that moral judgments rely on reasoning, either intuition or deliberation (Johnson-Laird, 2006; Bucciarelli et al., 2008). Surprisingly, there are no studies confronting the predictions of these theories in a population of children; they could enforce the assumptions of one of these theories with respect to the other.

In the present investigation, following the assumptions of the model theory, I make a critical prediction that contrasts with the assumptions of the socio-intuitionist and the moral grammar theories: children are more utilitarian than adults when dealing with the classical moral dilemmas.

The paper is organized as follows. First, I introduce the main assumptions of the socio-intuitionist theory, the moral grammar theories and the model theory. Second, I present a mental model account of reasoning upon classical moral dilemmas, then I describe two experiments involving children, adolescents and adults whose scope was to test the predictions deriving from the proposed account. In particular, as all but one participant in the adults groups of the two experiments were females, I tested model theory predictions for females. Finally, I discuss the results of the experiments: children's utilitarian judgments enforce the assumptions of the model theory.

## Current theories of moral judgment

Upon hearing that a woman donated a kidney to a friend who was suffering from serious kidney problems, saving him from certain death, one could experience an immediate positive emotion, while upon hearing that a woman suffering from HIV has voluntarily hidden her state of health from her new partner and spread the disease, one could experience an immediate negative emotion. Haidt (2001, 2007; see also Blair, 1995) proposes a *social-intuitionist* theory in which moral evaluations come from immediate intuitions and emotions. Thus, for example, the positive emotion experienced in relation to the kidney problem scenario would lead to judge the woman's donation as morally right, while the negative emotion experienced with respect to the HIV scenario would lead to judge the woman's behavior as morally wrong. Haidt argues that moral evaluations of this sort are fast, automatic and easy, based on an implicit perception of the problem as a whole, a process more akin to perception than reasoning (in line with Hume, 1978/1739), and calls them “moral intuitions,” Moral intuitions are “the sudden appearance in consciousness of a moral judgment, including an affective valence (good-bad, like-dislike), without any conscious awareness of having gone through steps of searching, weighing evidence, or inferring a conclusion” (Haidt, 2001, p. 818). For Haidt (2001, p. 814), “moral intuitions (including moral emotions) come first and directly cause moral judgments.” The social component of Haidt's theory postulates that conscious reasoning about moral issues comes only after intuitions about them, and that its role is to motivate moral judgments *ex post* and to affect the intuitions and as a consequence other people's moral evaluations. Contrary to intuitions, conscious reasoning occurs slowly, requires effort and includes at least some steps that are accessible to consciousness. Haidt admits the possibility of moral conflicts arising between intuitions; in that case, the final judgment will depend either on following the stronger intuition or on allowing deliberative reasoning to choose between the alternatives by applying rules or principles (Haidt, 2001). However, on rare occasions our moral evaluations are based on reasoning (see also Haidt and Graham, 2007). In Haidt's view, Kohlberg's results in favor of a role of reasoning in moral judgments must be interpreted in relation to his method of investigation. In particular, he used interviews that forced kids to explain complex notions such as how to balance competing concerns about rights and justice. As a result, their responses to the interview reflected the complexity of language rather than the complexity of the reasoning processes on which they based their moral evaluations. Haidt argues that a proper technique for testing children would be telling them short stories followed by single yes-or-no probe questions. The developmental prediction implicit in Haidt's assumptions is that children, like adults, base their moral judgments on their emotional reactions.

A possibility in contrast with Haidt's proposal is that emotional reactions to moral scenarios are the *results* rather than the *cause* of moral judgments. This position is maintained by the moral grammar theories, which deny a role of emotions in moral judgments. In particular, Mikhail (2000, 2011) and Hauser (2006a) assume the existence of a universal moral grammar, a series of principles operating at an unconscious level and guiding moral judgments. The principles are abstract, lacking specific content, and have nothing to do with emotions. Emotions follow from unconscious moral judgments. Although emotions may lead us to feel uneasy about our intuitions, they do not play a causal role in our moral judgments (Mikhail, 2011, but also Hauser, 2006b). Reasoning does not play a causal role either. The moral grammar theories develop an idea originally advanced by the philosopher Rawls (1971), who was the first to purport that a moral theory can be modeled on aspects of Chomsky's universal grammar (see Chomsky, 1986). Mikhail (2011), for example, purports that “ordinary individuals possess a complex moral grammar that enables them to judge the deontic status of actions in a manner roughly analogous to how native speakers intuitively recognize the grammaticality of sentences” (ib., p. 309). By the same analogy, as the universal grammar allows a native speaker of a language to evaluate whether or not a string of words corresponds to a sentence of the language, the moral grammar theories implicitly assume that it is always possible to evaluate an action as morally “right” or “wrong.” Consistent with the linguistic analogy, moral grammar theories assume that a child builds a particular moral system depending on the local culture which sets the parameters in a particular way (see Hauser, 2006a, p. 298). The resulting grammar automatically and unconsciously generates judgments of right and wrong for an infinite variety of acts and inactions. Some principles of the moral grammar are “transnational and possibly even universal or nearly so” (Mikhail, 2011, pp. 334–335). This conclusion, argues Mikhail, runs counter to one of the most basic assumptions by Piaget and Kohlberg according to which the adult's and the child's moral competence are comprised of fundamentally different principles. From the assumptions of the moral grammar theory descends the prediction that some principles guide children's moral judgments to the same extent they guide those of adults.

The model theory, contrary to the socio-intuitionist and moral grammar theories, argues that moral judgments rely on reasoning. The model theory, originally aimed to explain comprehension and reasoning (Johnson-Laird, 1983, 2006), has been extended to account for moral judgment (Bucciarelli et al., 2008). The theory makes two assumptions relevant to the present investigation, which are summarized in two principles.

***Principle of independent systems***. The principle postulates two independent systems, one handles emotions and the other handles reasoning, and they operate in parallel. According to this principle, the views that emotions can contribute to moral judgments (Greene et al., 2001; Haidt, 2001), and those implying that moral evaluations can contribute to emotions (Hauser, 2006b; Mikhail, 2011) are not correct. Bucciarelli et al. (2008) conducted an experiment and found evidence that there are scenarios, both moral and immoral, for which people either experience an emotion first, or make an evaluation first, or scenarios that are neutral in prevalence. Although moral judgments rely on reasoning, emotions can contribute to moral evaluations, and this may occur when, for instance, individuals reason about their emotions (Johnson-Laird et al., 2006).

***Principle of deontic reasoning***. The principle postulates that all deontic evaluations including those concerning matters of morality depend on inferences, either unconscious intuitions or conscious reasoning. In line with dual-process accounts of reasoning (for a review see Evans, 2010), the model theory purports the existence of two systems of reasoning, one fast, automatic and not subject to doubt, and the other slow, deliberate, and able to consider more than one option at the same time because it exploits working memory (Johnson-Laird, 2006). The former kind of reasoning is intuition (reasoning from unconscious premises to conscious conclusions); the latter kind of reasoning is deliberative reasoning (from conscious premises to conscious conclusions). Hence, differently from Haidt who argues that intuitions are contents of emotions, and from Mikhail and Hauser who argue that they are the product of a series of principles operating at an unconscious level, Johnson-Laird argues that intuitions are a form of reasoning. In particular, intuitions allow us to reason upon single possibilities, whereas deliberations allow us to reason upon multiple possibilities. In line with the assumption of the model theory that reasoning about moral propositions is unlikely to depend on a special process, and that it is merely normal deontic reasoning, neuroimaging studies have not detected areas in the brain specifically involved in moral judgments. Rather, several brain areas appear to offer important contributions to the production of a moral judgment (see Moll et al., 2008). Pascual et al. (2013) reviewed the main brain areas that have been associated with morality and observe that “the neural circuits of brain regions implicated in morality overlap with those that regulate other behavioral processes, suggesting that there is probably no undiscovered neural substrate that uniquely supports moral cognition.”

A third assumption relevant to my investigation is summarized in a fundamental principle of the model theory:

***Principle of parsimony***. The mind, in order to reduce the load on working memory, constructs salient mental models rather than complete mental models; salient models make explicit as little information as possible. Thus, when the reasoning task involves keeping multiple models in mind at once, we tend to think about them one at a time (Johnson-Laird, 2006, p. 203). As a consequence, we focus on that information which is explicit in our models and fail to consider other alternatives.

Legrenzi et al. (1993) argue that in many circumstances focusing implies that individuals will fail to make a thorough search for alternatives. In particular, if individuals are faced with the choice of either carrying out a certain action or not carrying it out, they will initially construct a model of the action and an alternative model in which it does not occur. The latter will be either implicit or else merely a model in which the action is negated. Thus, the choice between going to the cinema or not going to the cinema is represented by two disjunctive models. The first model is explicit and exhaustive, and so the other model, which corresponds to *not* going to the cinema, can be implicit:

[c]
…

where the rows in this schematic diagram represent two distinct possibilities: “c” denotes a model of going to the cinema and the parentheses indicate that the model is explicit and exhaustive, and the three dots denote the implicit model. Legrenzi et al. (1993) invited the participants in their experiment to gather information in order to be able to make a decision about whether or not to carry out a certain action and found that the participants tended to ask information about the action rather than possible alternatives. These results support the assumption that the participants were focused on the action to the exclusion of possible alternatives. Focusing should be reduced by any manipulation that makes alternatives to the action more available. In particular, the context can enable individuals to defocus by making alternatives available for fleshing out the implicit model. At this point, the participants can compare the attributes of the alternatives. A developmental assumption of the model theory is that children, because of their limited cognitive resources, are more likely than adults to construct and reason upon the model of single possibilities. The deriving predictions have been confirmed in both the factual (see Bara et al., 1995, 2001) and the deontic (see Bucciarelli, 2009) reasoning domains.

## How the theories account for moral judgments in moral dilemmas

The predictions of the socio-intuitionist theory, the moral grammar theories and the model theory have been confronted with respect to a set of moral dilemmas devised in the philosophical literature (see Foot, 1967; Thomson, 1986); some of these are known as “trolley problems.” The dilemmas involve two disjunctive possibilities. Consider, for example, the footbridge dilemma:

An empty boxcar is about to hit five people standing on the rail track, and it will kill them. You can push a man onto the track. Now, the boxcar will only hit that man and kill him, but the five people on the rail track will be safe.

Pushing the one person is: (permissible/impermissible).

When presented with this dilemma, adults tend to say that it is impermissible to kill one person to save five, while with the trolley dilemma, a modified version of the footbridge dilemma in which the killing of the one person occurs by redirecting the trolley onto a siding where the person stands, they produce a greater number of permissible responses (Greene et al., 2001; Cushman et al., 2006). The trolley dilemma reads as follow:

An empty boxcar is about to hit five people standing on the rail track, and it will kill them. You can pull a lever that sends the boxcar down another track. Now, the boxcar will hit and kill one man, but the five people on the rail track will be safe.

Pulling the lever is: (permissible/impermissible).

A utilitarian calculation of which option saves the most lives leads to a “permissible” response in both versions of the dilemma. Evidently, although adults tend to be more utilitarian with the trolley dilemma than with the footbridge dilemma, they do not base their moral evaluation on a utilitarian calculation.

According to the moral grammar theories, some principles of the moral grammar guide our judgments in moral dilemmas like the trolley problems. In particular, one of the principles holds that an otherwise prohibited action, such as battery or homicide, that has both good and bad effects, may be permissible if the prohibited act itself is not directly intended. Hence, the crucial difference between the footbridge dilemma and the trolley dilemma is that the former involves battery as the intended means: the choice is posited between committing a purposeful battery in order to prevent five people from dying; the trolley dilemma, instead, involves battery as a side-effect. Further, relevant to our investigation, Mikhail (2011) argues that “the distinction between intended means and foreseen side effect, are invariant throughout the course of moral development, at least between ages 8 and 65” (Mikhail, 2011, p. 349). From this assumption descends the prediction that young children should also find it more permissible to act with the trolley dilemma than with the footbridge dilemma. Pellizzoni et al. (2010) invited children aged 3–5 years to deal with both versions of the dilemma and found that they were more likely to judge it right to act in the trolley dilemma (pulling a cord) than in the footbridge dilemma (pushing a person: 88 and 25%, respectively). They concluded that children, like adults, conform to some of the principles postulated by the moral grammar theories and that such a continuity in moral cognition supports the existence of a domain-specific developmental mechanism rather than domain general mechanisms. Alas, their study does not exclude the possibility that the very young children in the experiment might have responded to the questions in the moral dilemmas regardless of the moral scenario to which the questions refer, namely merely focusing on the question. Hence, they might have responded affirmatively to the question “What is the right thing for Albert to do? Pull the cord or not pull the cord?” because “pulling a cord” *per se* is not an immoral action, and they might have responded negatively to the question “What is the right thing for John to do? Push the person or not push him?” because “pushing a person” *per se* is an immoral action. An unpublished study described by Mikhail (2011) has the same limit. The study was conducted on 30 children aged 8–12 years and utilized a between-subject design; each child encountered one of two moral dilemmas: in one battery was intended and in one it was a side effect. The task was to decide whether the proposed action was “wrong.” The results revealed that the children dealing with the battery as a side effect scenario were more likely to judge it right to act than the children dealing with the intended battery scenario (93% vs. 40% of the children, respectively). Also this study does not exclude the possibility that the children might have responded to the questions in the moral dilemmas merely focusing on the question. Consistent with this explanation, the production of “impermissible” judgments about the purposeful battery scenario was at chance level (only 9 out of the 15 children judged the action to be impermissible: Binomial test, *p* = 0.30, assuming a prior probability of 0.5). It is possible that the children in the study were too young to be able to comprehend the causal scenarios described in the dilemmas, a question I will return to when introducing the criteria used to select the experimental populations in my experiments. The proposed interpretation of the findings by Pellizzoni et al. (2010) and Mikhail (2011) is consistent with the so-called action aversion hypothesis: the condemnation of harmful actions, rather than deriving from considering outcomes, can be driven by an aversive response to the action itself (Miller et al., 2014; Patil, 2015).

Greene et al. (2001) advance a different explanation for adults being more utilitarian with the trolley version than the footbridge version of the dilemmas. He argues that a critical difference is that the footbridge dilemma involves physical contact with the victim but the trolley dilemma does not; killing without physical contact makes the dilemma “less personal” and therefore “less emotional,” so that the action to kill one person to save five more lives is deemed permissible. Utilitarian responses to personal dilemmas, instead, require the person to overcome an emotional response against inflicting direct harm on another person. The claim that emotions can affect our moral evaluations is consistent with the socio-intuitionist theory according to which emotions guide our evaluations. However, Greene et al. (2001, 2008) propose a different account from the socio-intuitionist theory and argue that in some circumstances reasoning guides our moral evaluations (see also Paxton and Greene, 2010; Paxton et al., 2012); whereas personal dilemmas elicit intuitive emotional responses that reflect concerns for right and duties, impersonal dilemmas engage controlled cognitive processes that support utilitarian judgments aimed at promoting the greater good. Consistent with this assumption, neuroimaging studies have revealed that brain areas associated with emotion are significantly more active while dealing with personal dilemmas than impersonal ones (Greene et al., 2001) whereas brain regions associated with cognitive control exhibit increased activity preceding utilitarian moral judgments made in response to personal moral dilemmas (Greene et al., 2004).

Moral dilemmas have been less investigated within the mental model framework. In one experiment by Bucciarelli et al. (2008; Experiment 4) the participants dealt with a series of dilemmas and carried out three tasks for each of them. First, they decided whether or not the action was permissible. Second, they modified the description of the dilemma so that they would switch their evaluation from permissible to impermissible, or vice versa. Third, they modified the description again to produce a version of the dilemma that they were unable to resolve. The results revealed that the participants readily modified dilemmas to switch their judgments from permissible to impermissible, and vice versa. Similarly, they were able to modify them still further to construct dilemmas that they would find impossible to resolve. When naïve individuals construct a new version of a dilemma, whether to switch an evaluation or to make it impossible for them to resolve, they also appear to be engaged in conscious reasoning. These results run against the existence of a moral grammar; the grammar is incompatible with the participants' ability to construct strings of sentences whose grammatical status is impossible for them to resolve. Bucciarelli and colleagues concluded that their results support the assumption that moral judgments are based on reasoning; when dealing with moral dilemmas individuals reason upon the mental models of alternative scenarios. This assumption has been endorsed by recent studies. In particular, Waldmann and Dieterich (2007) argue that individuals construct and reason upon the causal models of the alternative scenarios described in the dilemmas, and that the causal models have several features that can affect moral judgments. In particular, the dilemmas have different causal models depending on the locus of the intervention; either the intervention influences the path of the agent of harm (e.g., in the trolley dilemma) or the intervention influences the path of the potential patient (e.g., in the footbridge dilemma). The participants in the experiments dealt with four moral dilemmas including terrorist, medical and military settings. The results of the experiments revealed that an intervention which harmed a smaller number of people in order to save more people was more acceptable if the intervention targeted the agent rather than the patient. The overall results of the experiments suggest that a theory of moral reasoning based on causal mental models can account for some aspects of moral evaluations of moral dilemmas.

To sum up, the model theory, but not the socio-intuitionist or the moral grammar theories, assumes that reasoning plays a role in moral judgments.

## Reasoning upon moral dilemmas: A mental model account

Moral dilemmas present scenarios in which individuals have to choose between two alternative possibilities, to act or not to act, which both entail moral consequences. The classical moral dilemmas explicitly mention the possibility in which the action is performed, but not the one in which the action is not performed; thus, on the basis of the findings by Legrenzi et al. (1993), they focus individuals on such a possibility. Consider, for example, the footbridge dilemma: “An empty boxcar is about to hit five people standing on the rail track, and it will kill them. You can push a man onto the track. Now, the boxcar will only hit that man and kill him, but the five people on the rail track will be safe. Is it right that you push the man?.” The dilemma states that “pushing a man onto the track causes 1 person to die and 5 people are safe” (i.e., the possibility in which the action is performed), but it does not explicitly mention the possibility in which the action is not performed.

My experiment on moral dilemmas manipulated four variables that can foster “permissible” judgments. Indeed, besides killing a person as an unintended consequence, other features of the dilemma can foster “permissible” judgments (see Moore et al., 2011; Christensen et al., 2014):

No physical contact between the agent and the victim.
The action saves oneself along with others.
The agent is a third party, not the participant in the experiment.

First, the moral dilemmas in my experiment manipulated the fact of whether killing someone was an intended or an unintended consequence. Second, they manipulated the fact of whether or not there was physical contact between the protagonist and the victim. Third, they manipulated the fact of whether or not acting meant saving oneself along with others. Fourth, they manipulated the fact of whether or not the agent was a third party, not the participant in the experiment. As a result, the anti-permissible version of the trolley dilemma in my experiment is:

An empty boxcar is about to hit five people standing on the rail track, and it will kill them. You can push a man onto the track. Now, the boxcar will only hit that man and kill him, but the five people on the rail track will be safe. Is it right that you push the man?

The pro-permissible version of the trolley dilemma is:

An empty boxcar is about to hit and kill you and four other people standing on the rail track. Frank can pull a lever that sends the boxcar down another track, where it will kill one man, but you and the four people will be safe. Is it right that Frank pull the lever?

The assumptions of both the socio-intuitionist and moral grammar theories lead to predict that children, as well as adolescents and adults, are affected by the experimental manipulation and are more utilitarian with the pro-permissible versions of the dilemmas. According to the socio-intuitionist theory the pro-permissible versions are less emotional than the anti-permissible versions and, therefore, are more likely to lead both children, adolescents, and adults to utilitarian judgments. According to the moral grammar theories the pro-permissible versions differ from the anti-permissible versions in terms of universal principles that are at work from very early on in the scenario (i.e., no physical contact with the victim and killing as an unintended consequence vs. physical contact with the victim and killing as an intended action); on these grounds children, adolescents and adults should judge it more permissible to act on utilitarian grounds with the pro-permissible version of the dilemmas.

**Figure 1:**
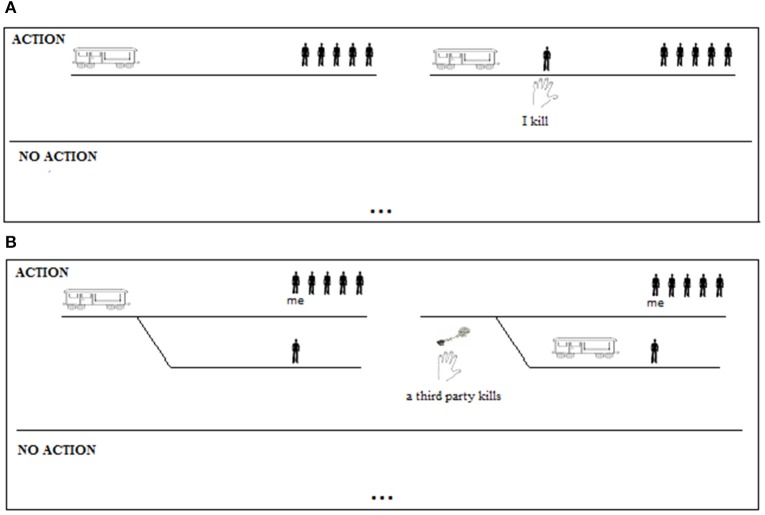
**The mental models of the possibilities in the anti-permissible (A) and pro-permissible (B) versions of the dilemma**. The diagram in each figure represents the alternative possibilities on opposite sides of a continuous line: the possibility in which the action is performed is represented through an explicit model; the alternative possibility, in which the action is not performed, is not represented (implicit models, represented by dots).

On the contrary, within the proposed mental model account adults but not children should be affected by the experimental manipulation; only individuals capable of deliberative reasoning can be affected by the manipulation. The mental models of the anti-permissible version of the dilemma represent the possibility of one person being killed intentionally by the participant in the experiment, with physical contact with the victim, and the life of the participant in the experiment does not depend on the action being performed (see Figure 1A). The mental models of the pro-permissible version of the dilemma represent the possibility of one person being killed unintentionally, by a third party rather than by the participant in the experiment, without physical contact with the victim, and the life of the participant in the experiment does depend on the action being performed (see Figure 1B).

In line with Legrenzi et al. (1993), I assumed that the manipulation of the four variables introduced above would affect individuals' judgments through mechanisms of focusing and de-focusing: saving oneself along with others strengthens the focus on the model representing the action, whereas being the agent, intentional killing and physical contact with the victim de-focus from the possibility of the action being performed, leading the participant to make explicit the model representing the possibility of the action not being performed. In particular, consistent with the assumption of the model theory that emotions can contribute to moral evaluations, when the participants in the experiment consider the possibility in which they are the agent and kill through physical contact with the victim, the emotions experienced with respect to this possibility may lead them to make explicit the model representing the possibility in which the action is not performed.

**Figure 2:**
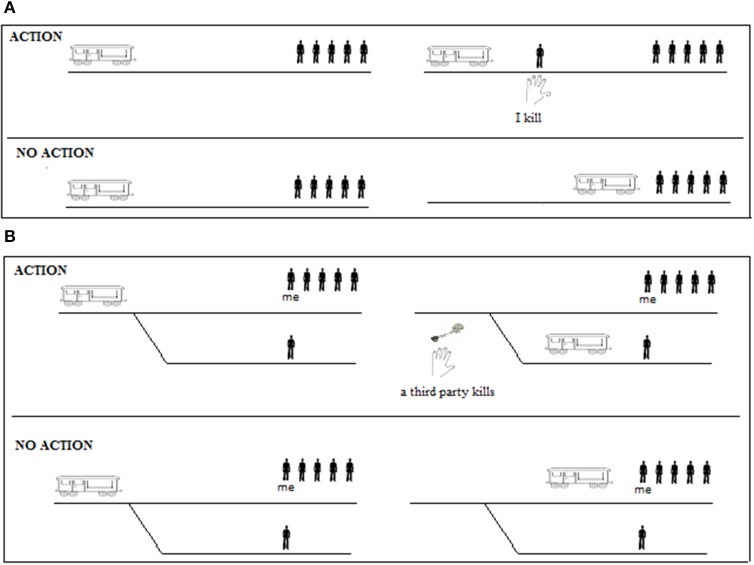
**The fully explicit mental models of the anti-permissible (A) and the pro-permissible (B) versions of the dilemma**. In each figure, the diagram represents the alternative possibilities on opposite sides of a continuous line.

I assumed that in the anti-permissible versions of the dilemmas focusing is reduced and de-focusing is increased, and in the pro-permissible versions of the dilemmas focusing is increased and de-focusing is reduced. From these assumptions derives the prediction that the anti-permissible version is more likely to lead reasoners to make fully explicit the alternative possibility in which the action is not performed. Figure 2 illustrates the fully explicit mental models for both versions of the dilemma.

The model theory for moral reasoning can easily be extended to account for children's moral judgments. Children construct and reason upon the model of single possibilities; hence, they rely on intuition, namely reasoning upon single possibilities. As children grow up their cognitive resources increase and they can rely on deliberation, namely reasoning upon multiple possibilities. From this assumption descends the prediction that children, more than adolescents and adults, are likely to focus on the model of the possibility mentioned in the premises of a dilemma, in which the action is performed, and to give utilitarian judgments in both the pro-permissible and the anti-permissible versions of the dilemmas. The prediction for adolescents is that they should be affected by the experimental manipulation more than children and less than adults. Adults should be affected by the experimental manipulation and be more utilitarian with the pro-permissible version of the dilemmas than with the anti-permissible version; in the latter case individuals are more likely to construct the explicit models of the dilemma and therefore be able to compare the attributes of the alternative possibilities, a process that can result in either utilitarian or non-utilitarian judgments. Hence, in my proposed framework utilitarian responses can be grounded on intuitions, i.e., reasoning upon single possibilities, as well as on deliberations, i.e., reasoning upon multiple possibilities. In both cases, they are signs of reasoning.

To sum up the predictions deriving from my assumptions:

*Prediction 1*: Children reason upon utilitarian grounds regardless of the manipulation, thus justifying the means with the ends in both the pro- and the anti-permissible versions of the dilemmas.

*Prediction 2*: Adolescents and adults are affected by the manipulation and are more utilitarian with the pro-permissible versions of the dilemmas.

*Prediction 3*: With the increase in age individuals are affected by the experimental manipulation: utilitarian responses to both versions of dilemmas decrease, but the decrease in utilitarian responses is greater for the anti-permissible versions of the dilemmas. Thus, the difference between the number of “permissible” responses to the pro-permissible version of the dilemmas as compared to the anti-permissible version increases.

This research was approved by the Bioethic Committee of Turin University. I tested my predictions on fifth-grade children, adolescents aged 13–14 years, and adults. These age groups have been found to differ in terms of cognitive abilities and resources supporting reasoning abilities (see, e.g., Bara et al., 1995). Children and adolescents took part in the experiment after their parents had given their informed consent.

## Experiment 1: Female children are more utilitarian than adults with moral dilemmas

The participants in the experiment encountered two versions of a series of six dilemmas. In each dilemma acting meant killing one person in order to save five others. I created two “extreme” versions for each dilemma: a pro-permissible version and an anti-permissible version. The pro-permissible version was less personal and less emotional than the anti-permissible version; hence, for the socio-intuitionist theory as well as for the dual process theory of Greene et al., children as well as adolescents and adults should be more likely to judge it permissible to act. At the same time, the pro-permissible version and the anti-permissible versions differed on the basis of several principles of the moral grammar; the deriving prediction, also in this case, was that children, adolescents and adults should be more likely to judge it more permissible to act in the pro-permissible version. On the contrary, from the assumption that individuals reason upon moral dilemmas and children are more likely than adolescents and adults to reason intuitively upon single possibilities, descends the prediction that the pro-permissible versions should foster “permissible” judgments in adults, but not in children. The two extreme versions of the dilemmas were meant to increase the participants' sensitivity to their differences, a procedure that runs against the assumption underlying the investigation, according to which children are not sensitive to such differences and are therefore more utilitarian than adults. The participants in the children and in the adolescents groups were balanced by gender, whereas those in the adult group were not: they were all females but one. Hence, the experiment tested the prediction that the pro-permissible versions of the dilemmas should foster “permissible” judgments in female adults, but not in female children.

For explorative purposes, the participants were also invited to explain their choice. Although the justifications may reflect post hoc rationalizations, it was interesting to see whether individuals tended to justify their choice by appealing to cognitive reasons rather than emotional factors (Bucciarelli and Daniele, 2015). The principle of deontic reasoning postulates that all deontic evaluations including those concerning matters of morality depend on reasoning. This assumption implies that in deontic decisions reasons are factors that motivate decisions and play a more important role than emotional factors; the experimental evidence is in favor of this assumption (see Shafir et al., 1993; Green et al., 1999, respectively). The same considerations hold for moral decisions; hence, when invited to explain their choice, the participants, children included, should appeal to reasons rather than emotional factors.

## Methods

### Participants

The aim of the experiment was to compare children's, adolescents' and adults' moral judgments: for this reason it was necessary to assure that the participants in the different age groups were homogeneous in terms of their social background. Also, the experimental task required them to read and comprehend the dilemmas: hence, it was necessary to exclude any individuals with cognitive disabilities or dyslexia from the sample.

Forty-two children in each of the following age groups were randomly selected from the middle class pupils of two junior schools in Turin, Italy: 9–10 years (21 females and 21 males; mean age 9;7 years, *sd* = 0.24), 13–14 years (21 females and 21 males; mean age 13; 6 years, *sd* = 0.28), and forty-two adults (41 females and one male; mean age 21 years, *sd* = 3.16). Children were selected on the basis of their teachers' assessment that they had no cognitive disabilities or dyslexia. The home background of the children attending the junior schools, which were state schools situated in the city center, suggested that they were likely to go to university. The adolescents attended state high schools commonly considered to be an intermediate step toward university. The adults were university students attending a course in general psychology at the University of Torino. They volunteered to take part in the experiment in exchange for course credits.

### Design

The moral dilemmas in the experiment were created from six contents; one pro-permissible version and one anti-permissible version was created for each content, for a total of 12 dilemmas.

Each participant encountered the two versions of the six moral dilemmas in separate blocks, so that the two versions of the same dilemma never occurred in the same block. In the overall group of participants a version of a dilemma occurred equally often in the first block and in the second block, and the dilemmas in each block were presented in random order. Two non-moral scenarios were used between the first block of dilemmas and the second block in order to prevent the possibility of two versions of the same dilemma being encountered in consecutive trials.

### Materials and procedure

The experiment was carried out in Italian. The full set of dilemmas is provided in Appendix A. An example of a dilemma in the pro-permissible version is:

You and four swimmers are drowning. George can drive a motorboat toward you at top speed. He will cause a passenger to fall into the sea, but he will save all of you. The passenger will drown because he cannot swim, but you and the four swimmers will be safe.

Is it right that Giorgio drive at top speed ? (Yes/No)

The corresponding anti-permissible version is:

Five swimmers are drowning. You can drive a motorboat toward them at top speed and save them if you lighten your boat. You can do that by pushing one of your passengers into the sea. He will drown because he cannot swim, but the five swimmers will be safe.
  Is it right that you drown your passenger? (Yes/No)

The experiment consisted of a single session that was carried out individually in a quiet room, in the sole presence of the experimenter. Each dilemma was printed on a sheet of paper, and the sheets were assembled in a booklet, in random order. The participants were instructed as follows: “This is an experiment on how people make decisions. I am going to present you with 14 scenarios and for each scenario your task is to decide whether it is right or wrong to perform a certain action. You have no time limit. Furthermore, as soon as you reach a decision please explain your choice.” The participants wrote their decision below each scenario. The entire experimental session was audio-recorded.

### Coding and statistical analyses

The “permissible” judgments in the two versions of the dilemmas were analyzed in terms of frequencies. The normality assumption was checked using the Kolmogorov-Smirnov test. The frequencies of the “Yes, it is permissible” responses by the three groups of participants in the experiment were in actual fact, not normally distributed. The Kolmogorov-Smirnov test determined that the frequencies of “permissible” responses to both the anti-permissible and the pro-permissible versions of the dilemmas did significantly differ from the normal distribution [KS-test: df(42), *d* varied from 0.19 to 0.31, *p* varied from < 0.001 to < 0.0001]. Statistical analyses were thus performed using non-parametric statistical tests. The Cliff's Delta statistic was used; such a non-parametric effect size measure quantifies the amount of difference between two groups of observations beyond *p*-values interpretation. The statistic provides information about the magnitude of the difference between the two groups of observations. If different ages of the participants in the experiment (independent variables) produce a statistically significant difference in the production of “permissible” responses to the two versions of moral dilemmas (dependent variables), the statistic quantifies such discrepancy. Cliff's delta runs from -1 to +1. +1 means that all of the values of one group are higher than all the values of the other, and -1 means the opposite. 0 means that the distributions are perfectly overlapping. Generally, it is just reported the absolute value, so Cliff's delta effectively ranges from 0 to 1.0. Assuming this, Romano et al. (2006) created these general guidelines for effect sizes with Cliff's delta: negligible (0.00 < delta < 0.14), small (0.14 < delta < 0.33), medium (0.33 < delta < 0.47) and large (0.47 < delta < 1.0).

In the Results section are summarized the crucial statistical comparisons.

If *Prediction 1* holds true (children reason upon utilitarian grounds regardless of the manipulation, thus justifying the means with the ends in both the pro- and the anti-permissible versions of the dilemmas) then in the children group there should be no statistically significant difference between the number of “permissible” responses to the anti-permissible versions of the dilemmas and those to the pro-permissible versions.

If *Prediction 2* holds true (adolescents and adults are affected by the manipulation and are more utilitarian with the pro-permissible versions of the dilemmas) then in the groups of adolescents and adults the number of “permissible” responses to the pro-permissible versions of the dilemmas should be greater than the number of “permissible” responses to the anti-permissible versions of the dilemmas and the difference should be statistically significant.

If *Prediction 3* holds true (with the increase in age individuals are affected by the experimental manipulation: utilitarian responses to both versions of dilemmas decrease, but the decrease in utilitarian responses is greater for the anti-permissible versions of the dilemmas) then there should be statistically significant trends in decrease of production of “permissible” judgments from children to adolescents till adults with both versions of the dilemmas, but there should be a statistically significant increase, from children to adolescents till adults, in the difference between the number of “permissible” responses to the pro-permissible versions of the dilemmas as compared to the anti-permissible versions.

The justifications provided by the participants for their decisions were transcribed then coded independently by two independent judges, blind to the aim of the experiment. They were scored according to the following categories:

*Cognitive reasons*, either reasons such as justifying the means by reference to the ends (utilitarian justifications) or reasons rejecting or questioning such appeals (non-utilitarian justifications). Examples of utilitarian protocols are: “Yes, in order to save more people, which is better than saving just one” (S1, children), “Yes, because although that person is innocent it is better that one dies in place of five” (S14, adolescents). Examples of non-utilitarian protocols are those in which the cognitive reason is a moral principle (e.g., “It is wrong to kill”), the actor's ignorance of consequence (e.g., “The person who will act does not know that he is going to kill one person”), and discrediting the consequence (e.g., “There is no reason why he should die because the five people shouldn't be on the train track”).

*Emotional factors*: the participant's emotions with respect to the means (e.g., “It would make me feel bad”).

*Both cognitive reasons* (either utilitarian or non-utilitarian) *and emotional factors* (e.g., “I wouldn't be able to push the person who would be an innocent victim”).

*Null justifications* (e.g., repetition of what is stated in the dilemmas; proposal of alternative solutions).

The two judges were instructed as follows: “A participant might produce a justification in which she refers to rational thoughts, personal opinions, norms and beliefs; this would be a ‘cognitive reasons’ justification. There are two different types of cognitive reasons; if they include achieving a good outcome for the greater number of people, they are utilitarian, otherwise they are non-utilitarian. Also, a participant might produce a justification in which she refers to emotional states, moods, and sentiments such as fear, shame, happiness. These emotional elements must be explicitly mentioned in the justification; this would be an ‘emotional factors’ justification. Finally, the participant might produce a justification in which she refers to both rational thoughts, personal opinions, norms, beliefs and emotional states, moods, and sentiments; this would be a ‘cognitive reasons and emotional factors’ justification.”

In the Results section are summarized the crucial statistical comparisons. They concern the percentages of type of justifications provided by the participants when invited to explain their choice. If, as implied by the assumptions of model theory, reasons are factors that motivate decisions and play a more important role than emotional factors, then the justifications appealing to cognitive reasons should be more than those appealing to emotional factors and the difference should be statistically significant.

## Results

Table 1 shows the percentage of responses in which the participants gave a “Yes, it is right” response (hereafter “permissible response”) for the two versions of the dilemmas. Appendix A shows the results for each dilemma in the two versions: anti-permissible and pro-permissible.

**Table 1:** **The percentage of responses in which the participants gave a “permissible” response for the two versions of the dilemmas in Experiment 1**.

	**Anti-permissible version**	**Pro-permissible version**
	**(*n* = 6)**	**(*n* = 6)**
Children	75%	80%
(*N* = 42)	(189/252)	(201/252)
Adolescents	36%	66%
(*N* = 42)	(91/252)	(166/252)
Adults	19%	49%
(*N* = 42)	(47/252)	(124/252)

*The balance of the percentage were “No, it's impermissible” responses*.

The crucial prediction was confirmed (Prediction 1): children were not affected by the experimental manipulation: the production of “permissible” judgments was comparable in the two versions of the dilemmas (Wilcoxon test: *z* = 0.88, *p* > 0.250, Cliff's δ = 0.02).

Still in line with the prediction, adolescents and adults were affected by the experimental manipulation: they gave more “permissible” judgments in the pro-permissible version as compared with the anti-permissible version (Wilcoxon test: *z* = 4.82, *p* < 0.001, Cliff's δ = 0.51, and *z* = 3.99, *p* < 0.001, Cliff's δ = 0.50, respectively) (Prediction 2). Adolescents and adults did not differ in terms of production of “permissible” judgments in the pro-permissible version of the dilemmas (in comparison with the anti-permissible version: Mann-Whitney test on the difference between “permissible” judgments in pro-permissible and anti-permissible version: *z* = 0.15, *p* > 0.250, Cliff's δ = 0.31).

**Figure 3:**
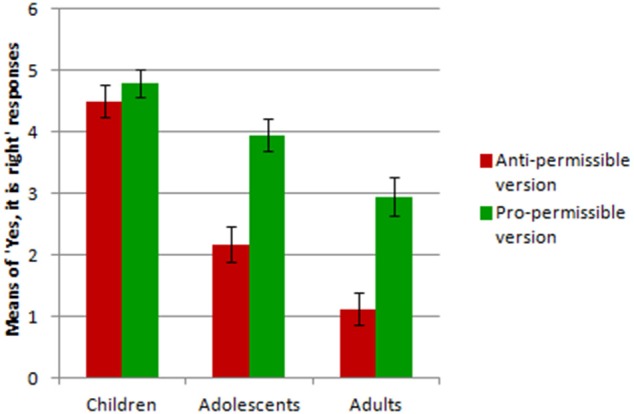
**The means of “Yes, it is right” responses to the two versions of the dilemmas by the three groups of participants in Experiment 1**.

Figure 3 presents the means of “Yes, it is right” responses to the two versions of the dilemmas by the three groups of participants. The developmental prediction was also confirmed (Prediction 3): in the anti-permissible version of the dilemmas the production of “permissible” judgments decreased from children, to adolescents to adults (Kruskal-Wallis test: *x*^2^ = 41.09, *p* < 0.001), and the Bonferroni-corrected *post-hoc* comparisons revealed differences between all the three age groups (p ranging from < 0.02 to < 0.0001). The same result held for the pro-permissible version of the dilemmas: the production of “permissible” judgments decreased from children, to adolescents to adults (Kruskal-Wallis test: *x*^2^ = 16.44, *p* < 0.001) and the Bonferroni-corrected *post-hoc* comparisons revealed differences between all the three age groups (p ranging from <0.03 to <0.05).

Further, the difference between the number of “permissible” responses to the pro-permissible version of the dilemmas and the anti-permissible version increased with age (Jonckheere's trend test over the three age groups of differences between each subject's number of “permissible” responses to pro-permissible version minus number of “permissible” responses to anti-permissible version: *z* = 3.58, *p* < 0.001). Finally, the participants in each age group differed reliably in giving “permissible” judgments in the 12 dilemmas (Friedman non-parametric analysis of variance, χ^2^ ranging from 37.74 to 122.87, *df* = 11, *p* always <0.0001).

A detailed analysis of the pairs of dilemmas revealed that for all pairs adults chose “permissible” more for the pro-permissible versions than for the anti-permissible versions. But, no such uniformity was observed among the children. This suggests that they were not sensitive to the difference between the pro- and anti-versions of the dilemmas. This, in turn, suggests that they did not really think very hard in order to make their decision. This result supported the assumption that children base their evaluations on intuitions rather than on deliberations.

There are three possible confounds in the experiment. (1) Differently from the other dilemmas, in dilemma 2 the potential victims were children. (2) The participants in the adults group were not balanced by gender. (3) The agent in the anti-permissible version of the dilemmas was neutral whereas in the pro-permissible version he was always a male. Three series of statistical analyses were conducted to exclude where possible these confounds.

***In dilemma 2 the potential victims were children***. A detailed analysis of the pairs of dilemmas revealed that the participants in the experiment were particularly prone to judging it permissible to act with dilemma 2. As a matter of fact, dilemma 2 differed from the others in that the potential victims were children. The overall results, according to which children were more utilitarian than adults, could be due to the fact that in dilemma 2 children were more likely to identify themselves with the potential victims and judge it permissible to act in order to save them. This possibility can be excluded: the difference between children's and adults' performance on dilemma 2 was smaller than for the other dilemmas. Children gave 86 and 90% of “permissible” judgments in the pro-permissible and the anti-permissible versions of dilemma 2, respectively, and 79 and 72% of “permissible” judgments in the pro-permissible and anti-permissible versions of the other dilemmas, respectively. Adults gave 69 and 48% of “permissible” judgments in the pro-permissible and the anti-permissible versions of dilemma 2, respectively, and 45 and 13 of “permissible” judgments in the pro-permissible and the anti-permissible versions of the other dilemmas, respectively. A comparison between children and adults revealed that the difference in performance with the two versions of the dilemmas was smaller for dilemma 2 than for the other dilemmas (Mann-Whitney test on the difference between “permissible” judgments in the pro-permissible and anti-permissible versions of dilemma 2 minus the difference between “permissible” judgments in the pro-permissible and anti-permissible versions of all the other dilemmas, and the same difference for adults: *z* = 3.19, *p* < 0.001, Cliff's δ = 0.39). Thus, the inclusion of dilemma 2 in the analyses of the results of the experiment ran against, rather than in favor of, the main prediction of the experiment.

***The participants in the adults group were not balanced by gender***. The participants in the adults group were all but one females. Recent findings have suggested that males are more utilitarian than females (Friesdorf et al., 2015); if this was the case in our experimental samples, then children resulted more utilitarian than adults simply because almost all of the adults were females. This possibility can be excluded: the results when considering only the females in the three groups of participants replicate the overall results. Table 2 shows the percentage of responses in which the female participants in Experiment 1 gave a “permissible response” for the two versions of the dilemmas. The crucial prediction was still confirmed (Prediction 1): children were not affected by the experimental manipulation: the production of “permissible” judgments was comparable in the two versions of the dilemmas (Wilcoxon test: *z* = 0.30, *p* > 0.250, Cliff's δ = 0.11). Adolescents and adults were affected by the experimental manipulation: they give more “permissible” judgments in the pro-permissible version as compared with the anti-permissible version (Wilcoxon test: *z* = 3.37, *p* < 0.001, Cliff's δ = 0.44, and *z* = 4.61, *p* < 0.0001, Cliff's δ = 0.55, respectively) (Prediction 2). Adolescents and adults did not differ in terms of production of “permissible” judgments in the pro-permissible version of the dilemmas (in comparison with the anti-permissible version: Mann-Whitney test on the difference between “permissible” judgments in pro-permissible and anti-permissible version: *z* = 0.41, *p* > 0.250, Cliff's δ = 0.03).

**Table 2:** **The percentage of responses in which the female participants gave a “permissible” response for the two versions of the dilemmas in Experiment 1**.

	**Anti-permissible version**	**Pro-permissible version**
	**(*n* = 6)**	**(*n* = 6)**
Children females	75%	79%
(*N* = 21)	(95/126)	(100/126)
Adolescent females	33%	60%
(*N* = 21)	(42/126)	(76/126)
Adult females	17%	49%
(*N* = 41)	(43/246)	(120/246)

*The balance of the percentage were “No, it's impermissible” responses*.

The developmental prediction was also confirmed (Prediction 3): in the anti-permissible version of the dilemmas we observed a decrease in the production of “permissible” judgments from children, to adolescents to adults (Kruskal-Wallis test: *x*^2^ = 29.58, *p* < 0.0001), and the Bonferroni-corrected *post-hoc* comparisons revealed differences between children and adolescents and between children and adults (*p* < 0.001 and < 0.0001, respectively), but not between adolescents and adults (*p* = 0.13). Also, in the pro-permissible version of the dilemmas we observed a decrease in production of “permissible” judgments from children, to adolescents to adults (Kruskal-Wallis test: *x*^2^ = 10.24, *p* < 0.006) and the Bonferroni-corrected post-hoc comparisons revealed differences between children and adults (*p* < 0.001), but not between children and adolescents and children and adults (*p* = 0.08 and 0.25, respectively).

Further, the difference between the number of “permissible” responses to the pro-permissible version of the dilemmas as compared to the anti-permissible version increased with age (Jonckheere's trend test over the three age groups of differences between each subject's number of “permissible” responses to pro-permissible version minus number of “permissible” responses to anti-permissible version: *z* = 3.03, *p* < 0.001). Finally, the participants in each age group differed reliably in giving “permissible” judgments in the 12 dilemmas (Friedman non-parametric analysis of variance, χ^2^ ranging from 22.67 to 123.54, *df* = 11, *p* varied from < 0.02 to < 0.0001).

Two non-parametric ANCOVA analyses where the effects of gender was removed by entering gender as a covariate revealed that the main effect of group still persisted for utilitarian responses to the anti-permissible version of the dilemmas [Quade's test: *F*_(2, 123)_ = 28.69, *p* < 0.0001] and for utilitarian responses to the pro-permissible versions [Quade's test: *F*_(2, 123)_ = 6.35, *p* < 0.002]. However, having entered gender as a covariate and accounted for variance associated with this variable, this only accounts for children and adolescents (which are gender-balanced anyway), but not for the adults group in which there is zero variance associated with males. Thus, the experimental predictions held for females and the results cannot be generalized to males.

***The agent in the anti-permissible versions was neutral whereas in the pro-permissible versions he was always a male***. If the participants in the experiment were likely to identify themselves with a same-gender agent in the scenarios, then they gave less “permissible” judgments in dilemmas in which the agent was of their same gender. As the agent in the anti-permissible versions was neutral whereas in the pro-permissible versions he was always a male, this possible same-gender effect can be explored for children and adolescents, who were balanced by gender. In particular, we were able to verify whether the males in the children and adolescents groups were more likely to judge it impermissible to act with the pro-permissible versions of the dilemmas, where the agent was a male, as compared to the females. An analysis detailed for children revealed that females and males did not differ in terms of production of “permissible” judgments in the pro-permissible version of the dilemmas (79 and 80% of permissible judgments, respectively) in comparison with the anti-permissible version (75% of permissible judgments for both genders: Mann-Whitney test on the difference between “permissible” judgments in pro-permissible and anti-permissible version: *z* = 0.23, *p* > 0.250, Cliff's δ = 0.06). The same result held for the adolescents: females and males did not differ in terms of production of “permissible” judgments in the pro-permissible version of the dilemmas (60 and 71% of permissible judgments, respectively) in comparison with the anti-permissible version (33 and 39% of permissible judgments, respectively: Mann-Whitney test on the difference between “permissible” judgments in pro-permissible and anti-permissible version: *z* = 0.77, *p* > 0.250, Cliff's δ = 0.14).

The justifications of the participants in the experiment for their choices also concurred to uphold the assumptions of the model theory. The two independent judges who coded the participants' justifications for their choices agreed in their coding in 91% of cases (Cohen's *K* = 0.87, *p* < 0.0001). For the final score, they discussed each item on which they disagreed, until reaching a full agreement.

Table 3 shows examples of coding and the percentages of the different types of justifications by the three groups of participants. The protocols were retrospective, and so they cannot refute Haidt's position. Nevertheless, they revealed that children appealed to cognitive reasons more than emotional factors in justifying their decision. And indeed, if we do not consider null justifications and consider an a priori probability of producing a cognitive justification of 1/3, children (37 out of 42), along with adolescents (34 out of 42) and adults (27 out of 42), produced cognitive justifications more often than chance (significant proportions on binomial test: *p* always < 0.0007). As a general result, over all participants and over all dilemmas, 62% of the justifications referred to cognitive reasons, 7% to emotional factors, and 3% to both. The remaining 21% were null justifications, namely protocols in which the participants repeated the premises or proposed alternative solutions for exiting the dilemmas.

**Table 3:** **Examples of verbal protocols for the main categories of justifications and relative percentages in the three groups of participants in Experiment 1**.

**Type of justification**				**Example protocol**	**Example dilemma**
Cognitive reasons				Yes, I save more persons (S15, Adults)	An empty boxcar is about to hit five people standing on the rail track, and it will kill them. You can push a man onto the track. Now, the boxcar will hit only that man and kill him, but the five people on the rail track will be safe. Is it right that you push the man?
	Chi	Ado	Adu	No because I could go in prison (Children, S38)	
	67%	66%	57%		
	(337/503^*^)	(329/502^*^)	(288/504)	Yes, if the man is able to sacrifice himself (Adolescents, S30)	
Util.	70%	45%	32%		
	(236/337)	(149/329)	(93/288)		
Non-util.	30%	55%	68%		
	(101/337)	(180/329)	(195/288)		
Emotional factors				No because pushing a person against a jib shocks me, also to see the person who dies (Children, S1) No I do not want to be an assassin and bear on my conscience a death caused by myself. I wouldn't feel to do that (Adolescents, S22) Yes, although the worker may have a family, in that case I would feel sorry (Adolescents, S15)	The jib of a crane is moving and will kill five workers. You can push one worker against the jib. Now the jib will stop when killing the worker. The worker will die, but the other five workers will be safe. Is it right that you push the worker?
	Chi	Ado	Adu		
	7%	6%	8%		
	(34/503^*^)	(31/502^*^)	(41/504)		
Cognitive reasons plus emotional factors				I'm sorry for the 5 persons who will trample on the bomb, but if that was their destiny I don't want to be involved by killing one person in their place (Adolescent, S1)	There is a bomb on a country road. It is not yet exploded. If it will be trampled on, the bomb will exploded. If you push a person on the bomb, the person will die, but five people who are arriving and will trample on the bomb will be safe. Is it right that you push the person?
	Chi	Ado	Adu		
	2%	3%	5%		
	(9/503^*^)	(14/502^*^)	(23/504)		
Null justifications				No because I could advise them (Children, S9)	
	Chi	Ado	Adu		
	24%	25%	30%		
	(123/503^*^)	(128/502^*^)	(152/504)		

* *(One children did not justify one response, and one adolescent did not justify two responses)*.

Leaving aside null justifications, more justifications referred to cognitive reasons than to emotional factors or both cognitive reasons and emotional factors (Wilcoxon test: *z* = 9.49, *p* < 0.0001, Cliff's δ = 0.94, *z* = 9.73, *p* < 0.0001, Cliff's δ = 0.98, respectively). The same results hold when considering the three age groups separately. More justifications referred to cognitive reasons than to emotional factors or both cognitive reasons and emotional factors in children (Wilcoxon test: *z* = 5.39, *p* < 0.0001, Cliff's δ = 0.94, *z* = 5.65, *p* < 0.0001, Cliff's δ = 0.99, respectively), in adolescents (Wilcoxon test: *z* = 5.65, *p* < 0.0001, Cliff's δ = 0.96, *z* = 5.65, *p* < 0.0001, Cliff's δ = 0.98, respectively), and in adults (Wilcoxon test: *z* = 5.498, *p* < 0.0001, Cliff's δ = 0.92, *z* = 5.63, *p* < 0.0001, Cliff's δ = 0.97, respectively). Further, justifications appealing to cognitive reasons did not increase with age (Jonckheere trend test: *J* = 2934, *z* = 1.29, *p* = 0.099).

A subcategory of cognitive reasons justifications are those appealing to utilitarian considerations. In line with the results revealing that children are more utilitarian than adults, the analysis of the justifications revealed that that utilitarian justifications for “permissible” responses decreased with age: 62% for children, 52% for adolescents, and 41% for adults (Jonckheere trend test: *J* = 1820.50, *z* = 2.47, *p* = 0.007). This result suggests that children base their utilitarian judgment on utilitarian cognitive reasons.

## Discussion

The overall results of the experiment confirmed the prediction that with classical moral dilemmas female children are more utilitarian than female adults; as the participants in the adults group were not balanced by gender, at the moment the results cannot be generalized to males. The results are consistent with the model theory's assumption that children's difficulty in constructing the fully explicit mental models of the alternative possibilities leads them to be utilitarian regardless of the experimental manipulation. However, one could argue that children's utilitarian judgments in Experiment 1 might reflect a bias toward action rather than genuine reasoning upon a single possibility. The aim of Experiment 2 was to discard this possibility.

## Experiment 2: A bias to act cannot explain female children's utilitarian responses with moral dilemmas

The participants in the experiment encountered a series of pairs of dilemmas in which the choice was again between whether to act or not. In one version of the dilemmas the action was directed at sacrificing one person to save five (pro-utilitarian version) and in one version the action was directed to sacrificing five people in order to save one (anti-utilitarian version). Since acting means saving one person and killing five, if children reason they should decide not to act and simply consider the alternative model in which the action is negated; the decision can be made without constructing the fully explicit models of the alternative possibilities (see Legrenzi et al., 1993). In Experiment 2, like in Experiment 1, males were not properly represented in the group of adults, but in this case what is not allowed is a proper comparison between children's and adults' performance depending on gender. The experiment is apt to exclude the possibility that children, both females and males, are biased to act with moral dilemmas.

## Methods

### Participants

Twenty children aged from 9 to 10 years (10 females and 10 males; mean age 9;4 years, *sd* = 0.31) and 20 adults (19 females and 1 male; mean age 23 years, *sd* = 5.34) took part in the experiment. The children were randomly selected from the middle class pupils of a junior school in Turin, Italy, according to the same criteria adopted in Experiment 1. The adult participants were university students attending a course in general psychology and took part in the experiment voluntarily in exchange for course credits. None of the participants had taken part in Experiment 1.

### Material and procedures

The participants were presented with the pro-permissible dilemmas used in Experiment 1 and were invited to decide whether the action was right. However, unlike in the pro-permissible dilemmas in Experiment 1, this version did not include saving oneself along with others. The reason was to avoid that, as a result of the experimental manipulation, in the anti-utilitarian version of the dilemmas a possible choice was acting to kill oneself. Also, in dilemma 2, the five victims were no longer children, but adults, to make the dilemmas more homogeneous. Two versions of each of these dilemmas were created: one in which acting meant saving five people instead of one (action pro-utilitarian), and one in which acting meant saving one person instead of five (action anti-utilitarian) (see Appendix B for the full set of dilemmas). Six pairs of the following sorts of dilemmas were obtained:

Action pro-utilitarian:

An empty boxcar is about to hit five people standing on the rail track.
Franco can pull a lever that will send the boxcar down another track, where there is a man, the man will be killed, but the five people will be safe.
Is it right that Franco pull the lever? (Yes/No)

Action anti-utilitarian:

An empty boxcar is about to hit one person standing on the rail track.
Lina can pull a lever that will send the boxcar down another track, where there are five men, the five men will be killed, but that one person will be safe.
Is it right that Lina pull the lever? (Yes/No)

Each participant encountered all the moral dilemmas in two blocks, so that the two versions of the same dilemma never occurred in the same block. Two scenarios involving a non-moral choice were used between the first block of dilemmas and the second block to exclude the possible occurrence of the two versions of the same dilemma in consecutive trails. In the overall group of participants a version of a dilemma occurred equally often in the first block and in the second block. The task for each participant was to decide whether or not the action was right. The participants were instructed as follows: “This is an experiment on how people make decisions. I am going to present you with 14 scenarios and for each scenario your task is to decide whether it would be right or not to perform an action. You have no time limit.” The participants wrote their decision below each scenario.

### Coding and statistical analyses

The “permissible” judgments in the two versions of the dilemmas were analyzed in terms of frequencies. The normality assumption was checked using the Kolmogorov-Smirnov test. The frequencies of the “Yes, it is permissible” responses by the two groups of participants in the experiment were in part not normally distributed. The Kolmogorov-Smirnov test determined that the frequencies of “permissible” responses to the pro-utilitarian versions of the dilemmas did not significantly differ from the normal distribution [KS-test: df(20), *d* = 0.19 and 0.18, *p* = 0.14 and 0.05 in children and adults, respectively]. But the test determined that the frequencies of “permissible” responses to the anti-utilitarian versions of the dilemmas did significantly differ from the normal distribution [KS-test: df(20), *d* = 0.31 and 0.37, *p always* < 0.0001, in children and adults, respectively]. Statistical analyses were thus performed using non-parametric statistical tests.

In the Results section are summarized the crucial statistical comparisons. If children and adults are not biased to act when dealing with the moral dilemmas then the number of “permissible” responses should be greater in the pro-utilitarian versions compared to the anti-utilitarian versions and the difference should be statistically significant. Indeed, if children like adults reason when dealing with the dilemmas, they realize that acting in the pro-utilitarian versions means killing one person and saving five, whereas acting in the anti-utilitarian versions means saving one person and killing five.

## Results

The overall results revealed a significant production of “permissible” responses in the pro-utilitarian version of the dilemmas: an overall 60% as compared with an overall 10% for “permissible” responses in the anti-utilitarian version (Wilcoxon test: *z* = 5.25, *p* < 0.001, Cliff's δ = 0.82). An analysis by single age group confirmed the results in both children (60% vs. 10%: Wilcoxon test: *z* = 3.84, *p* < 0.0001, Cliff's δ = 0.86) and adults (60% vs. 10%: Wilcoxon test: *z* = 3.64, *p* < 0.001, Cliff's δ = 0.78). The participants differed reliably in giving “permissible” judgments in the 12 dilemmas: this result holds for children and adults (Friedman non-parametric analysis of variance, χ^2^ = 80.67 and 94.29, respectively, *df* = 11, *p* < 0.0001 in both cases).

There were two possible confounds in Experiment 2. (1) The participants in the adults group were not balanced by gender. (2) The agent in the pro-utilitarian versions of the dilemmas was always a male, and the agent in the anti-utilitarian versions of the dilemmas was always a female. Two series of statistical analyses were conducted to exclude where possible these confounds.

***The participants in the adults group were not balanced by gender***. An analysis of the results limited to the females in the experiment excluded the possibility that the females participants in Experiment 1 were biased to act. The analysis revealed a significant production of “permissible” responses in the pro-utilitarian version of the dilemmas: an overall 57% as compared with an overall 9% for “permissible” responses in the anti-utilitarian version (Wilcoxon test: *z* = 4.39, *p* < 0.0001, Cliff's δ = 0.77). An analysis by single age group confirmed the results in both children (55% vs. 10%: Wilcoxon test: *z* = 2.68, *p* = 0.007, Cliff's δ = 0.73) and adults (59% vs. 9%: Wilcoxon test: *z* = 3.53, *p* < 0.0001, Cliff's δ = 0.78). The participants differed reliably in giving “permissible” judgments in the 12 dilemmas: this result holds for children and adults (Friedman non-parametric analysis of variance, χ^2^ = 37.70 and 89.24, respectively, *df* = 11, *p* < 0.0001 in both cases).

***The agent in the pro-utilitarian versions of the dilemmas was always a male, and the agent in the anti-utilitarian versions of the dilemmas was always a female***. The aim of Experiment 2 was to discard the possibility that children had a bias to act. One could object to the conclusion that the results of Experiment 2 discard this possibility. Indeed, the agent in the pro-utilitarian versions of the dilemmas was always a male, and the agent in the anti-utilitarian versions of the dilemmas was always a female. If the participants in the experiment identified themselves with the agent in the scenarios, in particular the females with the female agent in the anti-utilitarian dilemmas and the males with the male agent in the pro-utilitarian versions of the dilemmas, then males would give less “permissible” judgments in pro-utilitarian versions than females, and females would give less “permissible” judgments in anti-utilitarian versions than males. An analysis by gender in the group of children revealed no interaction: females and males did not differ in terms of production of “permissible” judgments in the pro-utilitarian version of the dilemmas (55% and 65% of permissible judgments, respectively) in comparison with the anti-utilitarian version (10% of permissible judgments in both groups: Mann-Whitney test on the difference between “permissible” judgments in pro-utilitarian and anti-utilitarian versions: *z* = 0.54, *p* > 0.250, Cliff's δ = 0.14).

## Discussion

The results of the experiment ruled out the possibility that females children might be utilitarian merely because they were biased toward action in general. Yet, it is surprising that 10% of children's and adults' responses consisted in deeming it morally right to act in order to save one person in place of five. An analysis of the results by single dilemma (see Appendix B) shed light on this result. Such responses were most frequent with the anti-utilitarian version of dilemma 2, which differed from the other anti-utilitarian dilemmas in which the potential victims (the one person and the five people) would have died through the same means. In this dilemma, the one person would die by being burnt in the house, whereas the five people would die due to the breaking of the window. It is possible that the means by which the potential victims would have died affected the moral evaluation.

## General discussion

The main assumption underlying the present investigation was that making a moral decision in a moral dilemma involves the construction of the mental models of the alternative possibilities it describes: a model of the action (to sacrifice one person in order to save five people, a utilitarian decision) and an alternative model in which the action is not performed (five people die and one person is safe). When reasoning requires keeping multiple models in mind, we tend to think about them one at a time, and a focusing effect leads us to think about the model representing the possibility explicitly mentioned in the premises. Hence, when dealing with a moral dilemma individuals represent a fully explicit model of the possibility mentioned in the premises, in which the action is being performed. The manipulation of some variables involved in the alternative possibilities can lead individuals to focus on the model in which the action is performed and defocus from the alternative model, or to defocus from the model in which the action is performed and focus on the alternative model representing inaction. A precondition for the effectiveness of the manipulation is that the individual has the capacity to represent and bear in mind the models of the alternatives described; this capacity increases with age. The theory predicts that children are not affected by the experimental manipulation and always focus on the possibility mentioned in the premises, in which the action is performed. In other words, children are utilitarian regardless of the experimental manipulation of the alternatives in the dilemmas.

In Experiment 1, the alternatives in the dilemmas were manipulated to obtain two “extreme” versions: for both the socio-intuitionist theory and the moral grammar theories the pro-permissible versions should foster more utilitarian responses than the anti-permissible versions in children as well as in adolescents and adults. On the contrary, my assumption led to the prediction that children would not be affected by experimental manipulation, unlike adolescents and adults. As a consequence children are more utilitarian than adults. The results of Experiment 1 confirmed the prediction for females. On the basis of the assumption of model theory there is no reason to expect that the prediction do not hold also for males, but in order to generalize the present results to males it would be necessary to include more males participants in the adults group of the study. Experiment 2 excluded the possibility that females children in Experiment 1 were simply biased to act; when presented with dilemmas in which acting ran against utilitarian considerations, they judged it impermissible to act.

Within the proposed theoretical framework utilitarian judgments can be the result of intuition as well as of deliberation, both types of reasoning. This claim is inconsistent with that of Greene et al. (2001; 2004) according to which deontological judgments in moral dilemmas (refraining from harm) are fast and emotion-based, while utilitarian judgments (deciding to harm) are slow because they are the result of deliberative reasoning. However, consistent with my claim Christensen et al. (2014) conducted a study on moral judgment controlling four conceptual factors, among which the instructions given to the participant and the length and expression style of the dilemma, and found that “both deontological and utilitarian decisions can be made equally fast, and both to personal and impersonal dilemmas” (*ibidem*, p. 16).

The socio-intuitionist theory is not able to explain the results concerning children's judgments in moral dilemmas. The theory's assumptions led to the prediction that, when dealing with moral dilemmas, children, adolescents, and adults would be guided by their emotional reactions. But, if so, they should judge it impermissible to perform the action described in the anti-permissible versions of the dilemmas, in which the participants in the experiment are the agent, they kill intentionally, and killing occurs with physical contact with the victim thus making the dilemmas more personal and therefore “more emotional” (Greene et al., 2001). The theory might explain children's utilitarian responses by assuming that they do not empathize with the victims in a dilemma; indeed, studies have revealed that damage to the prefrontal cortex mediating emotional responses increases utilitarian moral judgments (Koenigs et al., 2007). But this explanation is rather implausible, as empathy can be observed in very young infants from the age of 6 months (Hay et al., 1981). Previous studies have revealed that adult individuals scoring high on measures of antisocial personality traits are more likely to endorse utilitarian solutions to dilemmas (Bartels and Pizarro, 2011); it would be possible, although difficult, for the socio-intuitionist theory to argue that children have higher antisocial personality traits than adults. Consistent with my assumption that mere emotions cannot resolve the dilemmas, a study by Nakamura (2013) revealed that differences in moral judgments in the trolley and the footbridge dilemmas have to be attributed to differences in rational processing rather than differences in emotional involvement. The participants in the study encountered a set of moral dilemmas used in Greene et al. (2001) distinguished between personal and impersonal dilemmas. Nakamura analyzed the correlation structure among the dilemmas through factor analysis and structural equation modeling. The results suggest that the difference between the two kinds of dilemmas is owing to the varying involvement of the rational reasoning process rather than the extent to which they engage the emotional process. Further, still consistent with the results of the present investigation, the findings reported by Royzman et al. (2011 Experiment 2) suggest that reasoning has a greater role than emotions in moral dilemmas. The participants in their experiment encountered two moral dilemmas where the protagonist was faced with two alternative courses of action: to perform or not incestuous sexual intercourse in order to prevent serious harm to another person. For each dilemma, the participants had to: (1) judge which course of action would be the morally right one to take, (2) imagine the two different courses of action the protagonist could take and rate their affective responses to each, (3) check which of the two courses of action available to the protagonist carries greater overall costs for all concerned. The results revealed that the relative costs associated with the alternative courses of action under consideration were a significant determinant of the moral judgment in the dilemmatic context. Hence, Royzman and colleagues concluded that the evaluation of moral dilemmas eliciting strong and conflicting emotions relies on moral rules which are not necessarily linked to strong emotions (see also Royzman et al., 2009). These findings support the assumption of the model theory that emotions and reasoning are two systems working in parallel (see also Gubbins and Byrne, 2014).

The moral grammar theories are not able to explain the results of the present investigation. Their assumptions led to the prediction that individuals of any age should be sensitive to the experimental manipulation of the two alternative possibilities in a moral dilemma; the variables physical contact/no physical contact with the victim, and killing as an unintended consequence/as an intended action, should affect individuals' performance because they reflect principles of a universal moral grammar at work from very early on in the development process. However, the results of the present investigation are in line with the predictions derived from the proposed extension of the model theory: the experimental manipulation did not affect children. Therefore, it is likely that the manipulations affect the reasoning process through universal cognitive mechanisms (on this line of argumentation, see also Cushman and Young, 2011), which exploit the ability to represent fully explicit mental models.

In conclusion, the results of the present investigation corroborate a prediction descending from the assumptions of the model theory but not from the assumptions of the competing theories: female children are more utilitarian than female adults. Also, the results support the theory's assumption that moral judgments mainly rely on reasoning rather than emotions or innate moral principles; they reflect general cognitive capacities, such as the ability to represent and to reason about multiple possibilities, which become more sophisticated with age and underlie moral judgments.

The present investigation has three main limits that future studies might overcome.

First, I assumed that children, unlike adults, would not be affected by experimental manipulation of the two alternative possibilities described in the moral scenarios. I constructed the experimental material running against my prediction: I needed to show that even when the two versions of the dilemmas differed on a series of dimensions so as to be “extreme” versions of the dilemmas, children were not sensitive to such manipulation. Hence, what is lacking in the present investigation is a focus on the possible different roles played by the different variables in determining focusing and de-focusing effects with respect to the possibilities described by the moral scenarios. Further studies are needed to explore the possible different contributions of the variables of the reasoning process investigated here.

The second limit of the present investigation is that the participants in the adults groups were not balanced by gender and, therefore, I tested the experimental predictions on females and the results cannot be generalized to males. A possible future step in this investigation would be the inclusion of more males in the adults group. Yet, a relevant result is that if we consider the groups of children and adolescents in which the participants were balanced by gender, children, both females and males, were more utilitarian even than adolescents. And, indeed, within the proposed theoretical framework there is no reason to expect that females and males differ in general cognitive capacities such as the ability to represent and reason about multiple possibilities: the cognitive mechanism has to be the same.

The third limit of the investigation is that the social background of the participants in the different age groups was controlled: they were middle-class individuals. It is possible that individuals are more or less likely to be sensitive to the variables manipulated in the present investigation depending on their social class and culture. The model theory for deontic reasoning is domain-general, but allows for the possibility of moral contents and contexts differing across cultures (see, Khemlani et al., 2010). In this sense, culture rather than gender could be the key to understanding differences in the result of the reasoning process since culture could concur to determine the content on which individuals are disposed to reason.

### Conflict of interest statement

The author declares that the research was conducted in the absence of any commercial or financial relationships that could be construed as a potential conflict of interest.
