# Supplementary material for: Moral dilemmas in females: children are more utilitarian than adults
Source: Front Psychol. 2015 Sep 8;6:1345. doi: 10.3389/fpsyg.2015.01345 (PMC4562243; doi:10.3389/fpsyg.2015.01345)
Supplement: Supplementary file 1 [file DataSheet1.DOCX]

**Appendix A.**

The percentages of ‘permissible’ responses to the dilemmas in Experiment 1. Versions .1 are anti-permissible and versions .2 are pro-permissible.

| Dilemmas | Children | Adolescents | Adults |
| --- | --- | --- | --- |
| **1.1** An empty boxcar is about to hit five people standing on the rail track, and it will kill them. You can push a man onto the track. Now, the boxcar will only hit that man and kill him, but the five people on the rail track will be safe. Is it right that you push the man? | 83 | 36 | 12 |
| **1.2** An empty boxcar is about to hit and kill you and four other people standing on the rail track. Frank can pull a lever that sends the boxcar down another track, where it will kill one man, but you and the four people will be safe. Is it right that Frank pull the lever? | 79 | 76 | 50 |
| **2.1** A house is burning and five children who are inside will die. You can get them out and save them by pushing a man through a window in order to break the glass. The man will fall out and die, but the five children will get out and they will all be safe. Is it right that you push the man? | 91 | 57 | 48 |
| **2.2** A house is burning and you and four children are inside and will die. Sam can get you all out by throwing a brick that will break open a window. The brick will kill a man standing at the window. The man will die, but you and the four children will be safe. Is it right that Sandro throw the brick? | 86 | 38 | 69 |
| **3.1** Five swimmers are drowning. You can drive a motorboat toward them at top speed and save them if you lighten your boat. You can do that by pushing one of your passengers into the sea. He will drown because he cannot swim, but the five swimmers will be safe. Is it right that you drown your passenger? | 55 | 21 | 7 |
| **3.2** You and four swimmers are drowning. George can drive a motorboat toward you at top speed. He will cause a passenger to fall into the sea, but he will save all of you. The passenger will drown because he cannot swim, but you and the four swimmers will be safe. Is it right that Giorgio drive at top speed ? | 64 | 41 | 29 |
| **4.1** There is a bomb on a country road. It has not yet exploded. If someone trample on it, the bomb will explode. If you push a person onto the bomb, the person will die, but five people who are arriving and will trample on the bomb will be safe. Is it right that you push the person ? | 74 | 33 | 17 |
| **4.2** There is a bomb on a road. If it will be trampled on it will explode. If Carl explodes the bomb by transmitting an  electronic signal a person who is passing near by will die. But you and the other four people who are arriving on the road will be safe. Is it right that Carl explode the bomb? | 76 | 48 | 43 |
| **5.1** The jib of a crane is moving and will kill five workers. You can push one worker against the jib. Now the jib will stop when it kills the worker. The worker will die, but the other five workers will be safe. Is it right that you push the worker? | 69 | 43 | 14 |
| **5.2** The jib of a crane is about to kill you and four workers. Luca can press a button that will change the direction of the jib. The jib will kill another worker, but you and the other four workers will be safe. Is it right that Luca push the button? | 88 | 71 | 41 |
| **6.1** A car driver loses the control of the car. He is going against five people who are standing of the side walk and will kill them. If you push a person under the car the car will stop. The person will die, but the five persons will be safe. Is it right that you push one person? | 79 | 33 | 14 |
| **6.2** A car without control is coming against you and other four people and will kill you. If Dino throw a tube on the road the car will change direction and it will hurt another person. The person will die, but you and the other four people will be safe. Is it right that Dino throw a tube? | 36 | 69 | 50 |

**Appendix B.**

The percentages of ‘permissible’ responses to the dilemmas in Experiment 2. Version Pro-U are pro-utilitarian response, versions Anti-U are anti-utilitarian response.

| ­­­­­­­­­Dilemmas | Children | Adults |
| --- | --- | --- |
| **1. Pro-U** An empty boxcar is about to hit and kill five people standing on the rail track. Frank can pull a lever that will send the boxcar down another track, where there is a man, the man will be killed, but the five people will be safe. Is it right that Frank pull the lever? | 50 | 77 |
| **1. Anti-U** An empty boxcar is about to hit one person standing on the rail track. Lina can pull a lever that will send the boxcar down another track, where there are five men, the five men will be killed, but that one person will be safe. Is it right that Lina pull the lever? | 9 | 4 |
| **2. Pro-U** A house is burning and five people who are inside will die Sandro can get them out by throwing a brick that will break a window. One man who is near the window will die, but the five people will be safe. Is it right that Sandro throw the brick?. | 54 | 68 |
| **2. Anti-U** A house is burning and one person who is inside will die. Marta can get them out by throwing a brick that will break a window. Five men who are near the window will die, but the one person will be safe. Is it right that Marta throw the brick ? | 27 | 27 |
| **3. Pro-U** Five swimmers are drowning. Giorgio can drive a motorboat toward them at top speed. He will cause a passenger to fall in the sea. The passenger will drown because he cannot swim, but the five swimmers will be safe. Is it right that Giorgio drive at top speed ? | 23 | 41 |
| **3. Anti-U** A swimmer is drowning. Chiara can drive a motor boat toward him at top speed. She will cause five passengers to fall in the sea. The five passengers will drown because they cannot swim, but the one swimmer will be safe. Is it right that Chiara drive at top speed? | 4 | 4 |
| **4. Pro-U** There is a bomb on a road. If it will be trampled on it will explode. If Carlo explodes the bomb by transmitting an  electronic signal a person who is passing nearby will die. But five people who are arriving on the road will be safe. Is it right that Carlo explode the bomb? | 50 | 41 |
| **4. Anti-U** There is a bomb on a road. If it will be trampled on it will explode. If Eva explodes the bomb by transmitting an electronic signal five people nearby will die, but one person who is arriving on the road will be safe. Is it right that Eva make explode the bomb? | 4 | 4 |
| **5. Pro-U** The jib of a crane is about to kill five workers. Luca can press a button that will change the direction of the jib. The jib will kill another worker, but the five workers will be safe. Is it right that Luca push the button? | 50 | 77 |
| **5. Anti-U** The jib of a crane is about to kill one worker. Paola can press a button that will change the direction of the jib. The jib will kill 5 workers, but the one worker will be safe. Is it right that Paola push the button? | 4 | 9 |
| **6. Pro-U** A car without control is going against five people and will kill them. If Dino throw a tube on the road the car will change direction and it will hurt another person. The person will die, but the five people will be safe. Is it right that Dino throw a tube? | 54 | 68 |
| **6. Anti-U** A car without control is going against and kill. If Anna throw a tube on the road the car will change direction and i twill hurt other five people. The five people will die, but the one person will be save. Is it right that Anna throw the tube? | 4 | 4 |
